# Supplementary material for: Evaluation of Safety, Tolerability and Pharmacokinetic Characteristics of SA001 and Its Active Metabolite Rebamipide after Single and Multiple Oral Administration
Source: Pharmaceuticals (Basel). 2023 Jan 16;16(1):132. doi: 10.3390/ph16010132 (PMC9862565; doi:10.3390/ph16010132)
Supplement: Supplementary file 1 [file pharmaceuticals-16-00132-s001.zip › pharmaceuticals-2069287-supplementary.pdf]

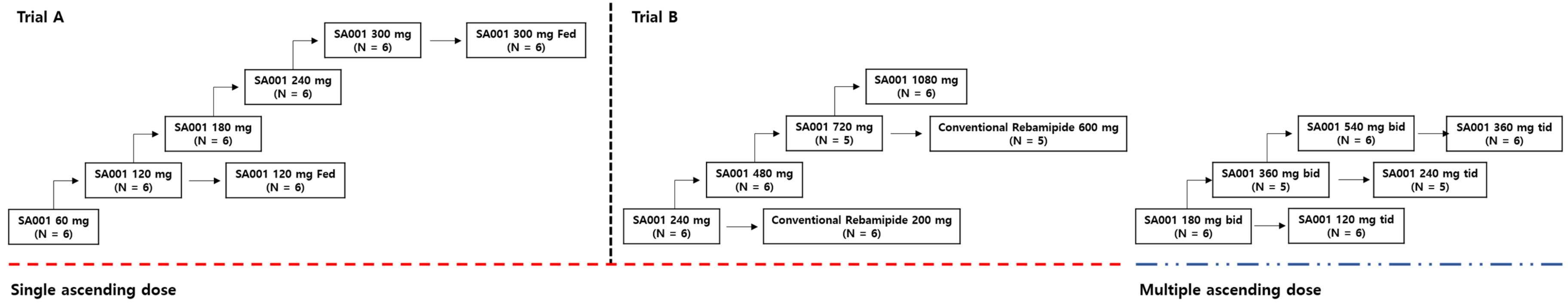

Supplementary Figure S1. Overall study design. Trial A was a single ascending dose (SAD) study. In 2 dose groups, pharmacokinetic (PK) characteristics of SA001 in the fed state were additionally analyzed. Trial B was composed of a SAD cohort and a multiple ascending dose (MAD) cohort. PK characteristics of SA001 and the conventional rebamipide were compared in 2 dose groups in the SAD cohort of Trial B. The MAD cohort of Trial B consisted of 3 dose groups. Subjects were initially administered half the assigned dose twice a day (b.i.d.) and then one third the assigned dose three times a day (t.i.d.) in the MAD cohort.

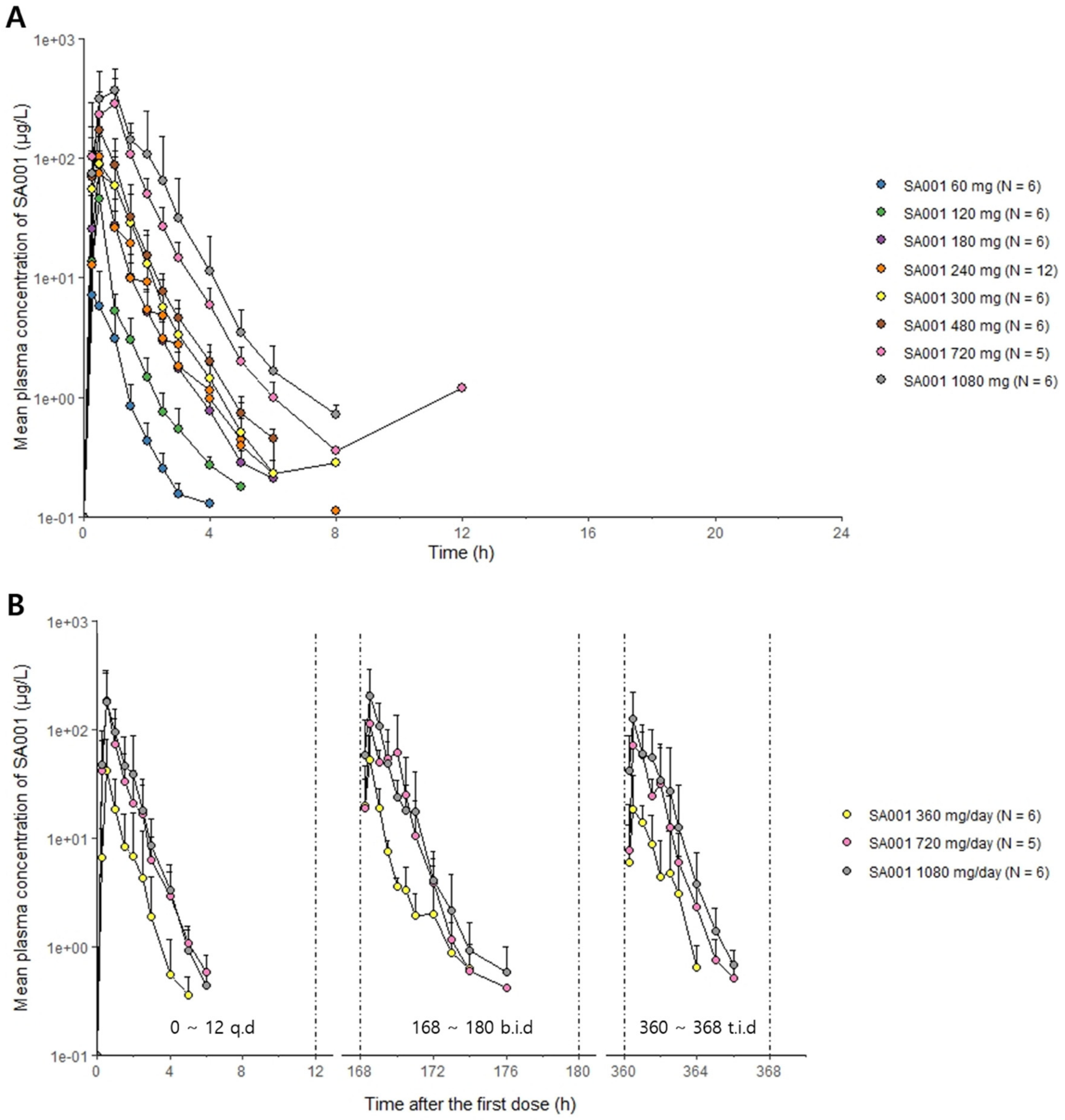

Supplementary Figure S2. Mean plasma concentration-time profiles of SA001 after (A) single and (B) multiple oral administrations of SA001 in log-linear scale. SA001 was administered twice a day (b.i.d.) with half the assigned dose until 180 hours after the first dose and three times a day (t.i.d.) with one third the assigned dose from 180 to 360 hours after the first dose in the multiple ascending dose cohort.

Bars represent standard deviations. Abbreviations: q.d; quaque die (once a day)

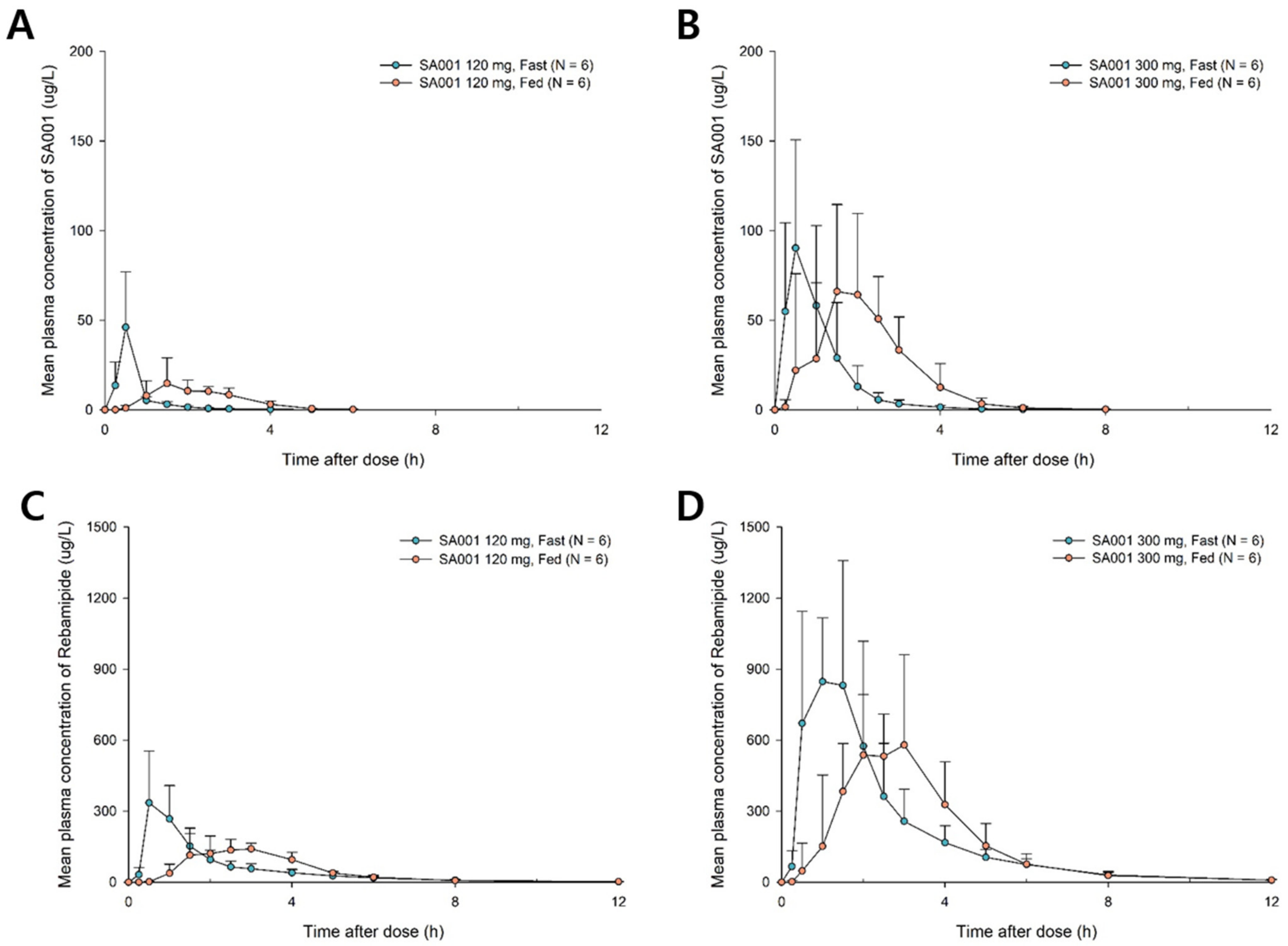

Supplementary Figure S3. Mean plasma concentration-time profiles of SA001 and rebamipide after single oral administration of SA001 in the fasted and fed state. (A) SA001 after administration of 120 mg of SA001 (B) SA001 after administration of 300 mg of SA001 (C) rebamipide after administration of 120 mg of SA001 (D) rebamipide after administration of 300 mg of SA001

Bars represent standard deviations.

Supplementary Table S1. Pharmacokinetic parameters of rebamipide after single oral administration of SA001 in the fasted and fed state

	Parameter	SA001 120 mg (N = 6)			SA001 300 mg (N = 6)		
		**Fasted state**	**Fed state**	**GMR * (90% CI)**	**Fasted state**	**Fed state**	**GMR * (90% CI)**
**SA001**	T_{max} (h)	0.5 (0.25, 1.5)	2.25 (1.5, 3)		0.5 (0.25, 1)	1.75 (0.5, 2.5)	
	AUC_{last} (ug*h/L)	22.66 ± 11.69	31.07 ± 10.82	0.6533 (0.4377 – 0.9571)	98.81 ± 19.57	148.59 ± 34.51	0.6685 (0.5830 – 0.7666)
	C_{max} (ug/L)	47.56 ± 28.89	19.37 ± 10.73	1.9747 (0.8266 – 4.7176)	127.28 ± 21.03	102.62 ± 31.33	1.2764 (0.9846 – 1.6545)
**Rebamipide**	T_{max} (h)	0.5 (0.5, 1)	2.5 (1.5, 3)		1 (0.5, 1.5)	2 (1, 3)	
	AUC_{last} (ug*h/L)	591.41 ± 265.58	503.45 ± 84.36	1.0803 (0.7953 – 1.4675)	2352.47 ± 700.48	2005.27 ± 392.26	1.1474 (0.8842 – 1.4891)
	C_{max} (ug/L)	344.94 ± 209.99	177.29 ± 42.56	1.6301 (0.9608 – 2.7656)	1148.74 ± 286.65	821.05 ± 276.68	1.4214 (1.0631 – 1.9005)

Notes: All values are presented as the arithmetic mean ± standard deviation. T_{max} values are represented as the median (minimum, maximum).

* Geometric mean ratio of the fasted to fed state.

Abbreviations: AUC_{last}; AUC from 0 to the time of the last measured concentration, C_{max}; maximum plasma concentration

Supplementary Table S2. Evaluation on dose linearity in the pharmacokinetic parameters of SA001 and rebamipide using the power model

Treatment	Parameter	Single administration		Multiple administration	
		**AUC_{last}**	**C_{max}**	**$AUC_{tau,ss}$**	**$C_{max,ss}$**
**SA001**	Intercept of Regression Line	-4.34	-2.92	-4.63	-3.16
	Slope of Regression Line (95% CI)	1.55 (1.38 – 1.72)	1.32 (1.13 – 1.52)	1.56 (1.35 – 1.78)	1.35 (1.02 – 1.67)
**Rebamipide**	Intercept of Regression Line	1.58	0.81	1.58	0.93
	Slope of Regression Line (95% CI)	1.04 (0.91 – 1.18)	1.07 (0.94 – 1.20)	1.07 (0.80 – 1.35)	1.08 (0.85 – 1.31)

Abbreviations: AUC_{last}; AUC from 0 to the time of the last measured concentration, C_{max}; maximum plasma concentration, $AUC_{tau,ss}$; AUC within a dosage interval (tau) at steady state, $C_{max,ss}$; maximum plasma concentration at steady state, CI; confidence interval

Supplementary Table S3. Summary of treatment emergent adverse events (TEAEs) by systemic organ class and preferred term after single oral administration of SA001

**System Organ Class** Preferred Term	**Placebo (N=18)**	**SA001 60mg (N=6)**	**SA001 120mg (N=6)**	**SA001 180mg (N=6)**	**SA001 240mg (N=12)**	**SA001 300mg (N=6)**	**SA001 480mg (N=6)**	**SA001 720mg (N=6)**	**SA001 1080mg (N=6)**	**Total (N=72)**
**Subjects with TEAEs**	**1(5.6) [1]**	**0**	**0**	**0**	**1(8.3) [1]**	**1(16.7) [3]**	**2(33.3)[2]**	**0**	**1(16.7) [4]**	**6(8.3) [11]**
**Gastrointestinal disorders**	**0**	**0**	**0**	**0**	**0**	**0**	**0**	**0**	**1(16.7) [1]**	**1(1.4) [1]**
Nausea	0	0	0	0	0	0	0	0	1(16.7) [1]	1(1.4) [1]
**General disorders and administration site conditions**	**0**	**0**	**0**	**0**	**0**	**1(16.7) [1]**	**0**	**0**	**0**	**1(1.4) [1]**
Pyrexia	0	0	0	0	0	1(16.7) [1]	0	0	0	1(1.4) [1]
**Injury poisoning and procedural complications**	**1(5.6) [1]**	**0**	**0**	**0**	**0**	**0**	**0**	**0**	**0**	**1(1.4) [1]**
Laceration	1(5.6) [1]	0	0	0	0	0	0	0	0	1(1.4) [1]
**Nervous system disorders**	**0**	**0**	**0**	**0**	**0**	**1(16.7) [1]**	**1(16.7) [1]**	**0**	**1(16.7) [2]**	**3(4.2) [4]**
Dizziness	0	0	0	0	0	0	0	0	1(16.7) [1]	1(1.4) [1]
Headache	0	0	0	0	0	1(16.7) [1]	1(16.7) [1]	0	1(16.7) [1]	3(4.2)[3]
**Respiratory, thoracic and mediastinal disorders**	**0**	**0**	**0**	**0**	**0**	**1(16.7) [1]**	**1(16.7) [1]**	**0**	**0**	**2(2.8) [2]**
Oropharyngeal pain	0	0	0	0	0	0	1(16.7) [1]	0	0	1(1.4) [1]
Rhinorrhea	0	0	0	0	0	1(16.7) [1]	0	0	0	1(1.4) [1]
**Skin and subcutaneous tissue disorders**	**0**	**0**	**0**	**0**	**1(8.3) [1]**	**0**	**0**	**0**	**1(16.7) [1]**	**2(2.8) [2]**
Acne	0	0	0	0	1(8.3) [1]	0	0	0	0	1(1.4) [1]
Urticaria	0	0	0	0	0	0	0	0	1(16.7) [1]	1(1.4) [1]

Notes: TEAEs are displayed as number of subjects(percentage of subjects) [number of events].

Supplementary Table S4. Relationship between the plasma concentration of SA001 and rebamipide with the corrected QT interval using linear regression

**Treatment**	**Parameter**	**Trial A**	**Trial B**
**SA001**	Mean of Slope of Regression Line (90% CI)	-0.0638 (-0.1408 - 0.0132)	-0.0077 (-0.0210 - 0.0055)
**Rebamipide**		-0.0077 (-0.0134 - -0.0021)	-0.0024 (-0.0042 - -0.0006)

Abbreviations: CI; Confidence interval
